# Abnormal electrocardiogram findings in athletes: A consensus statement of the European Association of Preventive Cardiology of the European Society of Cardiology

**DOI:** 10.1093/eurheartj/ehaf646

**Published:** 2025-09-25

**Authors:** Gherardo Finocchiaro, Alessandro Zorzi, Mark Abela, Aaron Baggish, Silvia Castelletti, Elena Cavarretta, Guido Claessen, Domenico Corrado, Maria Sanz de la Garza, Sabiha Gati, Viviana Maestrini, Aneil Malhotra, Josef Niebauer, David Niederseer, Michael Papadakis, Antonio Pelliccia, Sanjay Sharma, Flavio D’Ascenzi

**Affiliations:** Cardiovascular and Genomic Research Institute, City St George’s, University of London, Cranmer Terrace, London SW17 0RE, UK; Department of Cardio-Thoraco-Vascular Sciences and Public Health, University of Padova, Padova, Italy; University of Malta, Msida, MSD2090, Malta; Department of Cardiology, Mater Dei Hospital, Msida, Malta; Department of Cardiology, Lausanne University Hospital, Lausanne, Switzerland; Dipartimento di Cardiologia, IRCCS Istituto Auxologico Italiano, Milan, Italy; Department of Medical-Surgical Sciences and Biotechnologies, Sapienza University, Latina, Italy; Faculty of Medicine and Life Sciences, Biomedical Research Institute, LCRC, UHasselt, Hasselt, Belgium; Department of Cardio-Thoraco-Vascular Sciences and Public Health, University of Padova, Padova, Italy; Cardiovascular Institute, Hospital Clínic de Barcelona, Barcelona, Spain; School of Medicine, Imperial College London, London, UK; Institute of Sport Medicine and Science, National Italian Olympic Committee, Rome, Italy; Institute of Sport, Manchester Metropolitan University, Manchester, UK; Institute of Sports Medicine, Prevention and Rehabilitation, University Hospital Salzburg, Paracelsus Medical University, Salzburg, Austria; Hochgebirgsklinik Davos, Medicine Campus Davos, Davos, Switzerland; Cardiovascular and Genomic Research Institute, City St George’s, University of London, Cranmer Terrace, London SW17 0RE, UK; Institute of Sport Medicine and Science, National Italian Olympic Committee, Rome, Italy; Cardiovascular and Genomic Research Institute, City St George’s, University of London, Cranmer Terrace, London SW17 0RE, UK; Department of Medical Biotechnologies, Sports Cardiology and Rehab Unit, University of Siena, Siena, Italy

**Keywords:** Electrocardiogram, Sudden cardiac death, Sports cardiology

## Abstract

Athletes commonly exhibit a series of electrical, structural, and functional physiological changes which may overlap with cardiac pathology. The last two decades have witnessed a progressive improvement in understanding what can be considered benign for athletes and what may be deemed as potentially pathological and require further investigations. However, diagnostic uncertainties in the cardiac assessment of athletes are often encountered. In particular, the clinical significance of some electrocardiogram (ECG) findings may be uncertain. While uncommon and suggestive of an underlying cardiac condition, they may be identified among healthy athletes without additional pathological findings to support a unifying clinical diagnosis. This creates significant dilemmas for clinicians charged with determining sports eligibility and those who have the responsibility to help athletes in the decision-making process regarding future competitive sports participation. Current guidelines, recommendations, and position papers provide a roadmap for the differential diagnosis between ‘athlete's heart’ and cardiac disease. However, managing ECG findings of uncertain clinical significance, especially when initial diagnostic evaluation reveals no supportive signs of pathology, has received comparatively less attention, in particular, the type of cardiac investigations, the extent of diagnostic work-up and the need for follow-up require clarification. This document aims to provide guidance based on published evidence and expert opinions to assist in the clinical decision-making regarding ECG anomalies that are common sources of uncertainty when managing asymptomatic athletes.

## Graphical Abstract

**Graphical Abstract:**
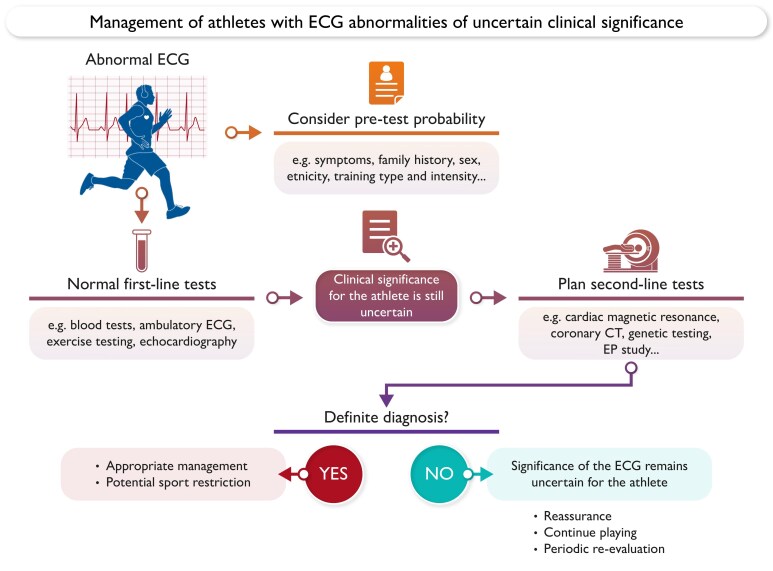
ECG interpretation in athletes presents several challenges due to physiological adaptations that can mimic pathological findings. A probabilistic approach is essential to refine pre-test probability, incorporating individual risk factors such as family history, sex, and ethnicity. When first-line tests fail to clarify the significance of ECG abnormalities, second-line investigations such as advanced imaging or functional assessments play a crucial role. In some cases, uncertainty may persist even with thorough diagnostic work. In such circumstances, the athlete may resume playing with periodic re-evaluation. Investigations shown are illustrative and not mandatory for all cases.

## Introduction

Cardiac conditions associated with an increased risk of sudden cardiac death (SCD) are often indicated or detected by abnormalities on a 12-lead electrocardiogram (ECG).^1–5^ However, interpreting ECGs in athletes can be challenging as these individuals often display a series of physiological, electrical, structural, and functional adaptations that overlap with cardiac pathology. While there is now a better understanding of the spectrum of athletic remodelling, including the ability to more accurately differentiate benign training-related ECG changes from those that indicate underlying disease,^6^ grey areas with corollary clinical uncertainty persist. Specifically, some ECG findings typically associated with underlying cardiac disease may be observed in healthy athletes without additional pathological findings to support a unifying clinical diagnosis. This poses significant challenges for clinicians tasked with assessing sports eligibility and engaging in shared decision-making about future competitive sports participation. Although current guidelines, recommendations and position papers, including the International recommendations for ECG interpretation in athletes, offer guidance for distinguishing between ‘athlete's heart’ and cardiac disease, the management of ECG findings deemed as abnormal, in the absence of other signs of pathology after comprehensive diagnostic evaluation, has not received adequate attention. Furthermore, certain ECG findings are not sufficiently tackled by the International recommendations and clarification regarding the type and extent of cardiac investigations is needed.

This document aims to provide clinical guidance based on published evidence and expert opinions to support clinical decision-making. While athletic grey zones can occur with all diagnostic tests, this document will specifically focus on those encountered on a 12-lead ECG. We will address isolated electrical abnormalities, where the term ‘isolated’ refers to findings suggestive of pathology that are not accompanied by a personal or family history suggestive of cardiac disease or the detection of pathological findings on first-line investigations. For the purpose of this document, we will use the term ‘athlete’ to define an individual who engages in competitive sports or intense regular exercise. The primary objective of this document is to provide guidance on the necessity and the type of clinical work-up for isolated ECG abnormalities and for ECG findings of uncertain clinical significance. It is important to note that further investigations should always be pursued when these anomalies are linked with other anamnestic or clinical abnormalities. The objective of this document is to reduce variability in care while allowing management strategies tailored to the unique requirements of each athlete. Additionally, we seek to provide clinicians with a pragmatic reference to guide their decision-making process.

## Methods

This consensus statement was proposed by the nucleus of Sports Cardiology of the European Association of Preventive Cardiology (EAPC) and approved by the Scientific Committee of the EAPC and the European Society of Cardiology (ESC) Scientific Document Committee in 2023. Current members of the EAPC Sports Cardiology nucleus and other distinguished sports cardiology experts were invited to participate as co-authors. Those who agreed were initially invited to an online meeting to discuss the aims of the project and to define ECG anomalies which should be discussed. Thereafter, each co-author was assigned to critically appraise a specific topic (ECG pattern) under the coordination of the chairs. A second meeting was held during the 2024 EAPC Congress in Athens to discuss the state of progress. Once the initial contributions were collected, a first draft was circulated together with an electronic survey for voting on the proposed recommendations. Three choices were offered: agree, agree with rephrasing, or disagree. The results of the poll were discussed during a second online meeting, after which the authors were asked to confirm whether they agreed or disagreed with the rephrased recommendations. According to the ESC policy on consensus statements, the strength of advice was classified into five categories on a scale from 0 to 4 bars. When a recommendation was based on robust evidence, the notion was designated 4 bars. When a recommendation was predominantly based on expert opinion, the notion was assigned 3 bars when all the authors agreed (‘uniform consensus’) while only 1 or 2 bars were assigned to a notion when >80% but not all experts were in agreement. The distinction between 1 bar and 2 bars was based on some published evidence or consensus opinion, respectively. Finally, when consensus could not be reached by at least 80% of authors, the statements were assigned an empty bar. After this process, a second draft was circulated and authors were offered the opportunity to provide further comments, which were handled by the chairmen. The document was subsequently forwarded to the EAPC Scientific Committee for internal blinded peer review. The manuscript was modified according to the reviewers’ comments and was finally approved by the EAPC Scientific Committee and the ESC Scientific Document Committee in 2024.

## Bradycardia and atrioventricular conduction abnormalities

### Marked sinus bradycardia (<30 b.p.m.)

Sinus bradycardia is observed in up to 80% of high-level athletes.^6^ Endurance athletes are more likely to develop sinus bradycardia, usually associated with myocardial remodelling. Heart rates below 35 b.p.m. are rare during waketime hours and typically occur only in extreme endurance athletes.^7^ Sinus arrhythmia, junctional rhythms, wandering pacemaker, sinus pauses, and atrioventricular (AV) conduction delays are present in up to 70% of bradycardic athletes.^6^ These benign arrhythmias are more frequently observed during sleep when the sinus rate may fall to below 30 beats/min.^8^

Vagal hypertonia has been extensively investigated as a causative mechanism of marked sinus bradycardia. Interestingly, even after complete pharmacological blocking of the autonomous nervous system using atropine and beta blockers, trained athletes still exhibit lower resting heart rates compared to sedentary people.^9^ Recent data suggests that intrinsic changes occur in the sinus node,^10^ which may persist after detraining. A longitudinal study in 157 former elite athletes with bradycardia (resting heart rate < 50 b.p.m. when they were engaged in high-level competitions) revealed that after >5 years of abstinence from training and competitions, 65% of participants had persistent bradycardia, with 18% exhibiting a heart rate < 50 b.p.m.^11^

In the absence of other clinical or electrocardiographic signs of sinus dysfunction, sinus bradycardia in an asymptomatic athlete should pose no clinical concern. In accordance with the International recommendations for ECG interpretation in athletes,^6^ a resting heart rate above 30 b.p.m. may be considered normal for asymptomatic athletes who engage in intense training and, thereby require no further examination. The exception would be athletes, particularly those over the age of 35, in whom the heart rate response during exercise is abnormal.

Among athletes with a heart rate < 30 b.p.m. at 12-lead ECG, with no or minimal chronotropic response despite a brief episode of brisk exercise or hyperventilation, further cardiac investigations are required. The clinical work-up should include basic blood tests (electrolytes and thyroid function test), transthoracic echocardiography (TTE), to rule out an associated structural heart disease, maximal exercise tolerance test (ETT), to assess chronotropic response and 24–48 h ambulatory ECG monitoring. The latter should incorporate a training session to demonstrate normal heart rate response and chronotropic reserve, and rule out sinus pauses or AV conduction abnormalities. It is important to note that marked bradycardia may be exacerbated by certain medications and is often associated with severe relative energy deficiency^12^ (e.g. in athletes with anorexia nervosa).^13,14^

Genetic testing may be considered to rule out mutations in HCN4, SCN5A, and ANK2, if marked bradycardia is not justified by intense training, is familial, or there is an association with structural heart disease.^15–18^ Despite the aforementioned cases, genetic testing is not recommended routinely in isolated ECG abnormalities, and may be considered only when supported by additional clinical or familial indicators. Invasive testing with an electrophysiology study has limited yield. However, it may be considered in carefully selected cases, for example, in cases with marked sinus bradycardia associated with persistent first-degree or with associate bundle branch block, exercise-unresponsive second-degree Mobitz I AV block, or suspected sinus node dysfunction presenting with unexplained exertional symptoms, despite a normal non-invasive evaluation.^19^

In the absence of symptoms or suspicious family history, an athlete with isolated marked sinus bradycardia and no other abnormalities should not be discouraged from engaging in high-intensity and/or high-volume sport. It is advisable to consider follow-up even in asymptomatic athletes, particularly if there is a discrepancy between the amount or the intensity of exercise and the degree of bradycardia (*Table 1*). When sinus node dysfunction is suspected, a short detraining period may be necessary to differentiate it from physiological bradycardia induced by exercise. An extended period of detraining (>6 weeks) is expected to be required to return to normal levels.^11,20^

**Table 1:** Diagnostic work-up of athletes with marked sinus bradycardia/AV conduction abnormalities and bundle branch blocks

Marked sinus bradycardia, AV block and short PR interval	
In athletes with marked sinus bradycardia, first-degree AV block with marked PR prolongation (>400 ms) or daytime second-degree Mobitz type I AV block that persists despite brisk exercise or hyperventilation, clinical investigations including basic blood tests, TTE, maximal ETT and ambulatory ECG monitoring, including a training session, are advised.	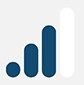
In athletes with marked PR prolongation or diurnal second-degree Mobitz type I AV block, testing for anti-Ro/SSA the athlete and the mother (in young athletes) may be considered.	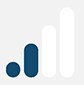
In athletes with first-degree or second-degree AV block associated to bundle branch block, a more comprehensive clinical work-up to exclude genetic, infiltrative or inflammatory diseases should be considered.	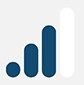
In athletes with AV block and associated bundle branch block and in symptomatic athletes with short PR interval and no overt pre-excitation, electrophysiological study may be appropriate.	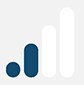
A detraining period of at least 2 months may be useful to differentiate between training-, marked first-degree AV block, or diurnal Mobitz type I AV block with normal initial investigations.	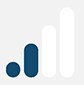

Bundle branch blocks	
In ostensibly healthy athletes presenting with LBBB, TTE is strongly advised as first-line examination to identify underlying structural heart disease and assess systolic function.	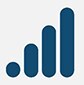
In athletes with LBBB, ETT and ambulatory ECG monitoring, including a training session, are advised to exclude AV conduction disease.	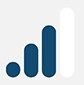
In athletes with LBBB, CMR is advised as a second-line test even if TTE is normal.	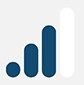
Coronary computed tomography angiography (CTCA) is advised as a second-line investigation in athletes with LBBB who are symptomatic or have a high risk profile for coronary atherosclerosis.	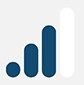
Genetic testing may be appropriate in selected athletes with LBBB, in the presence of structural abnormalities, AV block and/or atrial fibrillation.	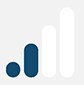
In athletes with RBBB and QRS duration < 130 ms, further investigations are not advised in those without a family history of premature cardiac disease or SCD, or concomitant ECG abnormalities.	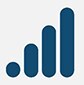
TTE deserves consideration in athletes with RBBB and QRS duration ≥ 130 ms.	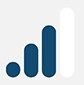
In athletes with complete RBBB and concomitant ECG abnormalities, it may be appropriate to perform a CMR, even if no apparent abnormalities are detected during TTE.	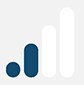
In athletes with complete RBBB, positioning V1 and V2 in the second and third intercostal spaces may be helpful to exclude a type I Brugada ECG pattern.	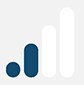

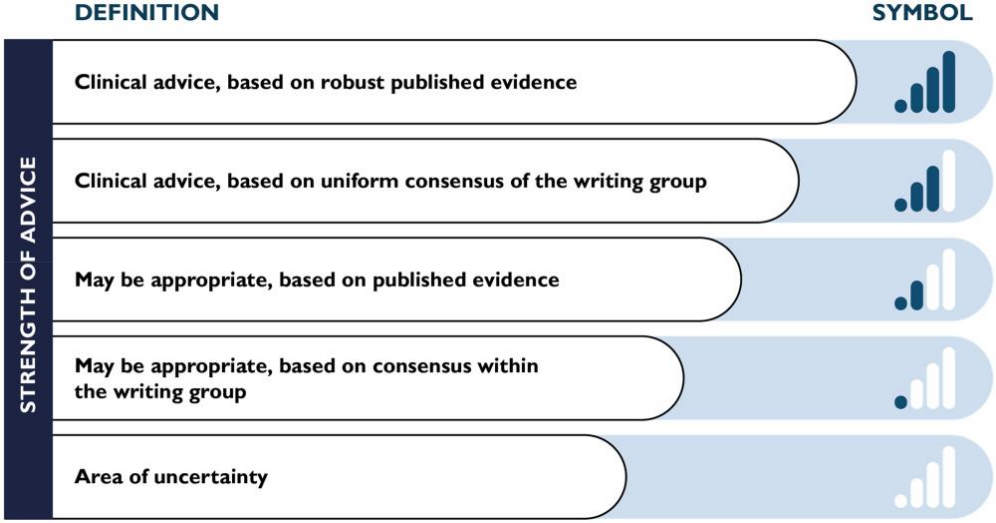
AV, atrioventricular; BrP, Brugada pattern; CCTA, coronary computed tomography angiogram; CMR, cardiac magnetic resonance; ETT, exercise tolerance test; LBBB, left bundle branch block; RBBB, right bundle branch block; Ro/SSA, anti-Sjögren's-syndrome-related antigen A autoantibodies; TTE, transthoracic echocardiogram; SCD, sudden cardiac death.

### First-degree AV block with marked PR prolongation (>400 ms)

First-degree atrioventricular block (AVB) is considered a common ECG finding in competitive athletes. Its prevalence increases with age (from 0.3% in children to 7.5% in elite adult athletes)^6,21^ and the PR interval prolongation is usually moderate (<250 ms). Adaptive first-degree AVB is typically due to increased vagal activity and intrinsic AV node changes.^7^ The PR interval typically shortens with the exercise stimulus and detraining. In contrast, a markedly prolonged PR interval (defined as ≥400 ms in adults and ≥300 ms in children/adolescents) is uncommon, even in elite athletes, and should prompt further investigation aimed to exclude an underlying heart disease, higher degree AVB and life-threatening arrhythmias.^22^ This is particularly important in the presence of coexisting bundle branch block, which should raise suspicion of infra-hissian block.

In athletes with markedly prolonged PR interval, which is persistent despite a brief episode of brisk exercise or hyperventilation, preliminary investigations should include a detailed family history, an ambulatory ECG monitoring, including a training session, TTE and ETT.^23^ It should be considered to investigate the autoimmunity status of the young athlete and the mother to identify potential congenital, late progressive congenital or acquired autoimmune AV block caused by maternal anti-Ro/SSA-autoantibodies (anti-Sjögren's-syndrome-related antigen A autoantibodies).^24–26^ It should be noted that anti-Ro/SSA testing may be considered in exceptional cases of unexplained or progressive conduction disease, but not routinely in asymptomatic first-degree AV block.

Second-line investigations are advised to exclude structural heart disease, infiltrative diseases and Lyme disease, particularly when the AV block is associated with bundle branch block or echocardiographic abnormalities. Among athletes with unexplained AVB with marked AV prolongation, in whom a potentially inherited cardiac disorder is suspected, genetic testing for mutations in *the SCN5A, TRPM4*, Lamin A/C and *PRKAG2*,^13,27,28^ and ECG screening of first-degree family members can be considered. Consideration should be given to performing an electrophysiological study in specific cases where non-invasive evaluation fails to characterize the origin of conduction disturbances definitively^29^ (*Table 1*).

Although participation in sports should not be impacted in asymptomatic individuals with forms of autoimmune AVB and a negative family history of SCD, follow-up is essential to evaluate the potential progression of the AV block. This also applies to athletes with a PR interval ≥ 400 ms in adults and ≥300 ms in children/adolescents and no other electrical and/or structural heart abnormalities.

### Daytime (‘diurnal’) second-degree Mobitz type I AV block

Physiological second-degree Mobitz type 1 AVB is commonly observed in athletes with a high vagal tone. Nocturnal Mobitz type 1 s-degree AVB and marked bradycardia are common in healthy athletes and do not warrant further investigation. Benign AVB in healthy athletes generally improves after mild exercise or even forced hyperventilation.^22^ In contrast, high-grade AVB or persistent Mobitz I AVB during the day and training sessions (i.e. ‘diurnal’ AVB) should raise suspicion of pathology. Although there are reported cases of second-degree Mobitz type 1 AVB due to infra-nodal block, most Wenckebach-type AVB are due to a transient and benign AV node conduction disturbance. Mobitz type 1 AVB is uncommonly caused by structural, infiltrative, infectious or genetic heart diseases (such as Lamin A/C cardiomyopathy, Lyme disease and cardiac sarcoidosis).^27,30–33^ In rare cases, autoimmune disorders may directly affect the AV node and manifest as Wenckebach-type AVB.^34^

Mobitz type I AVB should be investigated when it is associated with cardiac symptoms or relevant family history, persists during physical activity including vigorous exercise, is accompanied by wide QRS complexes, and/or other ECG abnormalities (atrial enlargement, axis deviation, ST-segment depression, T-wave inversion, low QRS voltages), or chronotropic incompetence during exercise.^6^

**Figure 1:**
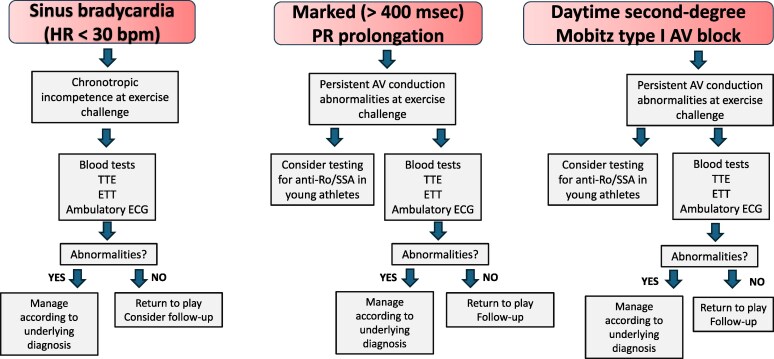
Management of athletes with bradyarrhythmias. It has to be noted that the recommendations on athletes with extreme bradycardia and marked PR prolongation apply to individuals in whom brisk exercise or hyperventilation does not rapidly resolve the anomaly. ETT, exercise tolerance test; HR, heart rate; TTE, transthoracic echocardiogram

Among those where the block fails to correct despite a period of brisk exercise or hyperventilation, an ETT is pivotal in evaluating chronotropic response and detecting a potential worsening of the AVB during progressive exercise (*Figure 1*). Additionally, continuous ambulatory ECG monitoring, including during training sessions, proves invaluable when assessing AV conduction throughout the day and night. Structural heart disease should initially be excluded with TTE. In specific scenarios, serological and genetic testing and cardiovascular magnetic resonance (CMR) may be helpful when considering autoimmune, infective or genetic aetiologies as described in the prior section for profound first-degree AV block^26^ In instances where the phenotype is unclear (e.g. worsening of the AVB during sympathetic stimulation or associated bundle branch block), consideration of an electrophysiological study to rule out infra-nodal disease may also be necessary^35^ (*Table 1*).

Finally, a detraining period may offer valuable insights by demonstrating diurnal AVB resolution and its correlation with symptoms.^20^

Asymptomatic athletes with diurnal Mobitz type 1 AVB that improves with a brief exercise period should not be precluded from engaging in sports.

### Short PR interval without overt pre-excitation (<120 ms)

A short PR interval (<120 ms) may indicate either accelerated conduction through the AV node or the presence of an accessory pathway (AP), thus leading to ventricular pre-excitation (manifesting as a widened QRS complex of >120 ms with a delta wave). A short PR without overt pre-excitation is not uncommon in athletes and observed in 0.6%–15.1% of children and adolescent athletes^36,37^ as well as non-athletes. Genetic factors may play a role in both athletes and non-athletes. The presence of a perinodal AP utilizing the ‘bundle of James’ has been suggested as a possible explanation for a short PR interval with no pre-excitation, although its existence remains a matter of debate.^38^

Electrophysiological study should be considered in athletes with a short PR interval who report palpitations suggestive of paroxysmal supraventricular tachycardia. An asymptomatic athlete with an isolated short PR interval in the absence of an overt delta wave or other ECG abnormalities does not require further evaluation.^6^

However, it should be noted that in athletes with an AP, the degree of pre-excitation generating the ‘delta wave’ on the ECG depends on the amount of myocardium that is activated through the AP and the AV node. In particular, when the AP is located in the mitral annulus distant from the sinus node, the impulse is preferentially conducted through the AV node, and therefore, a clear delta wave may be missing.^39^ Subtle ECG signs of pre-excitation include a tall R wave in V1, absence of Q wave in lateral precordial leads, left axis deviation, T-wave abnormalities and ST-segment depression during exercise.^40^ Slowing of the AV node conduction caused by vagal stimulation (during night-time or the recovery phase after exercise) may unmask the pre-excitation. When symptoms of palpitations are reported, and when in doubt, adenosine challenge may be helpful in this setting.

## Depolarization abnormalities and premature ventricular beats

### Left bundle branch block

Left bundle branch block (LBBB) is a rare occurrence in asymptomatic healthy individuals, with an estimated prevalence between 0.1% and 0.8%.^41^ Longitudinal data for LBBB in athletic cohorts are lacking, but studies in the general population indicate that LBBB is associated with increased cardiovascular morbidity and mortality in asymptomatic individuals.^42^ The five-year incidence of SCD as the initial manifestation of heart disease is 10 times greater in men with LBBB than in those without it.^42^ Therefore, LBBB is not part of the physiological adaptation to exercise and should always prompt further investigation. The specific components of this diagnostic evaluation are not firmly established and will depend on the clinical context.

**Figure 2:**
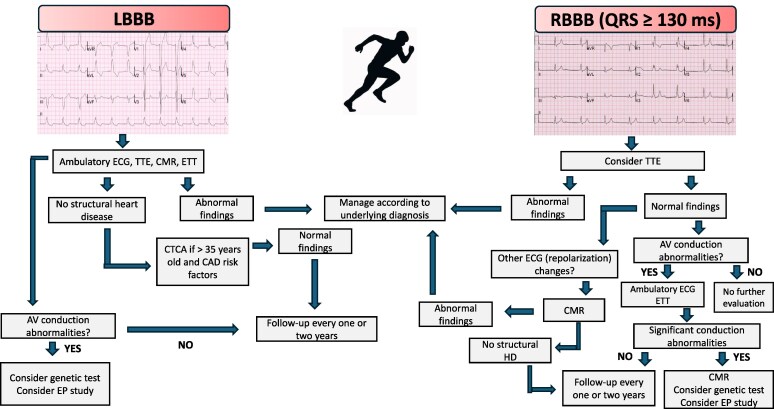
Management of athletes with LBBB and RBBB. AV, atrioventricular; CAD, coronary artery disease; CMR, cardiac magnetic resonance; CTCA, computed tomography coronary angiography; EP, electrophysiological; ETT, exercise tolerance test; HD, heart disease; TTE, transthoracic echocardiogram

In ostensibly healthy athletes presenting with LBBB, TTE is advised as a first-line examination to identify underlying structural heart disease and to assess the impact of dyssynchrony on myocardial systolic function.^43^ Additionally, ETT and ambulatory ECG monitoring should be considered to exclude the possibility of dynamic AV block. In asymptomatic athletes with LBBB and a normal TTE, CMR is advised as a second-line investigation, as this test can detect subclinical structural heart disease in asymptomatic patients with LBBB despite normal TTE results^44^ (*Figure 2*). The presence of LBBB alone is not an independent predictor of coronary artery disease in low-to-moderate-risk patients, but coronary computed tomography angiography (CTCA) should be considered as a second-line investigation depending on individual risk assessment, considering symptoms, demographics (age > 35 years and male sex), and risk factors for coronary atherosclerosis.^45^ When LBBB is associated with AV conduction abnormalities or abnormal features on cardiac imaging, a more in-depth clinical work-up, including genetic testing to rule out potentially serious heritable conditions (such as myotonic dystrophy, laminopathies, and progressive conduction disease), should be considered (*Table 1*).

If a comprehensive diagnostic work-up fails to produce a clear diagnosis, it is advisable to schedule follow-up assessment with an echocardiogram, every one or two years. When LBBB is accompanied by even mild structural and/or functional abnormalities suggestive of a heritable cardiac disease, screening of first-degree relatives appears appropriate.

### Right bundle branch block (with QRS ≥ 130 ms)

Complete right bundle branch block (cRBBB) is defined as a rSR′ pattern in lead V_1_ with a QRS duration ≥ 120 ms and an S wave wider than the R wave in lead V6. This finding is uncommon in the general population, with a reported prevalence of 0.1%,^46^ but it is reported in 0.2%–3% of competitive athletes.^47,48^ Among a cohort of 510 healthy competitive athletes, an isolated typical cRBBB (i.e. average QRS duration 125 ± 5 ms) was associated with a physiological increase in left ventricular mass and/or right ventricular chamber dimensions.^49^ When cRBBB is accompanied by other ECG findings, including atrial enlargement and QRS axis deviation, the likelihood of underlying heart disease increases (48).

In athletes with cRBBB, QRS duration should be taken into account for clinical management. A recent study involving 104 369 consecutive apparently healthy young individuals screened with an ECG and health questionnaire showed that cardiac disease was more common in subjects with non-isolated cRBBB with a QRS duration of ≥130 ms.^46^ In accordance with the International recommendations for ECG interpretation in athletes, additional diagnostic testing for athletes with an isolated cRBBB is not recommended.^6^ However, we suggest that in athletes with cRBBB and QRS duration of ≥130 ms TTE should be considered at least once to exclude an underlying structural cardiac disease (with a stronger mandate for athletes exhibiting cRBBB associated with other ECG abnormalities, given the low yield in isolated cRBBB^46^) (*Table 1*, *Figure 1*). Despite the low diagnostic yield of TTE in this setting, the rationale for this consideration is to exclude underlying structural heart disease, such as atrial septal defects, rather than to screen for conditions associated with a heightened risk of SCD.

Further investigations, such as CMR, may be necessary, even in the absence of obvious abnormalities at TTE, if other ECG changes are present, including precordial lead T-wave inversion involving the inferior and/or lateral TWI and marked QRS fragmentation. Additionally, recording of the V1 and V2 leads in the second and third intercostal space may be useful to differentiate RBBB from the coved-type (type 1) Brugada ECG pattern.^50^

Athletes with RBBB and QRS duration of ≥130 ms, in the absence of structural abnormalities or significant AV conduction disease, should not be restricted from sports participation. However, repeat assessment every 1 or 2 years is advised in athletes with coexisting abnormal or borderline electrical patterns.

### Isolated low voltages in limb and precordial leads

Low QRS voltages in the limb leads are defined as a QRS amplitude (from peak to nadir) < 0.5 mV in all the limb leads.^51^ Low QRS voltages in the precordial leads are defined as an amplitude < 1.0 mV in all precordial leads. Low QRS voltages isolated to the precordial leads are not addressed in this document due to the lack of clinical data pertaining to their significance.^51^ The prevalence of low QRS voltages in apparently healthy athletes is estimated to range between 1% and 2%,^52^ but this ECG pattern is non-specific and may be identified in a number of non-cardiac (obesity, hypertrophic pectoral muscles, emphysema, breast augmentation procedures and hypothyroidism) and cardiac (pericardial effusion, cardiomyopathies, infiltrative disease and myocardial scar)^53,54^ diseases. Specifically, low QRS voltages are more frequently present in patients with arrhythmogenic cardiomyopathy (ACM) with and without left ventricular (LV) involvement and isolated non-ischemic LV fibrosis (14%–22%) than in healthy athletes.^55–58^ Although this ECG feature may indicate an underlying structural heart disease that predisposes to an increased risk of SCD, its presence or significance is not discussed in the current international recommendations for ECG interpretation in athletes.

**Figure 3:**
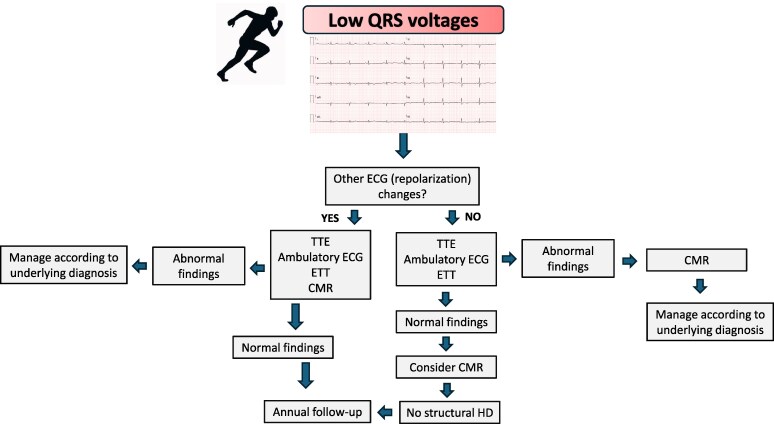
Management of athletes with isolated LQRSV. CMR, cardiac magnetic resonance; ETT, exercise tolerance test; HD, heart disease; TTE, transthoracic echocardiogram

Athletes with low QRS voltages and normal personal and family history should undergo TTE, maximal ETT and 12-lead ambulatory ECG monitoring, including a training session (*Table 2*, *Figure 3*). The latter two tests aim to identify premature ventricular contractions (PVCs) conducting with wide RBBB patterns (*Table 3*) that may suggest underlying non-ischemic LV fibrosis.^59^

**Table 2:** Diagnostic work-up of athletes with low QRS voltages in the limb leads and premature ventricular contractions

	Degree of recommendation
Among athletes with unexplained LQRSV in the limb leads, further investigation, including a TTE, maximal ETT and ambulatory ECG monitoring comprising a training session, is advised. CMR should be performed in athletes displaying abnormalities on these preliminary tests.	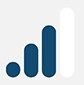
In athletes with LQRSV and negative initial tests, a CMR to exclude an underlying non-ischemic LV scar may be appropriate.	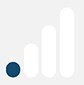
In athletes with one or more PVCs on the resting ECG, with the exception of fascicular PVC (with QRS <130 ms), 24-hour ambulatory ECG monitoring, preferably with 12-lead configuration, is advised, including a training session.	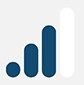
In athletes with high-risk features PVCs (*Table 3*) and/or with a PVC burden > 10% and/or with associated ECG abnormalities, further investigations are advised including blood tests^a^, TTE, maximal ETT and CMR.	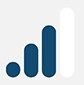
In athletes without high-risk PVCs (*Table 3*), associated ECG abnormalities or PVC burden > 10%, but with PVC burden > 1% and <10%, further investigation with a TTE is advised, and if this test is normal, follow-up should be considered.	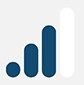
Genetic testing for CPVT is advised in athletes with exercise-induced, polymorphic PVCs suggestive of CPVT (e.g. increasing in frequency and complexity with adrenergic stimulation), in the context of a structurally normal heart.	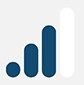
Invasive evaluation with electrophysiological study with programmed ventricular stimulation and endocardial voltage mapping may be appropriate in selected cases, with symptoms, high ectopic burden and complex PVCs.	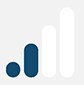

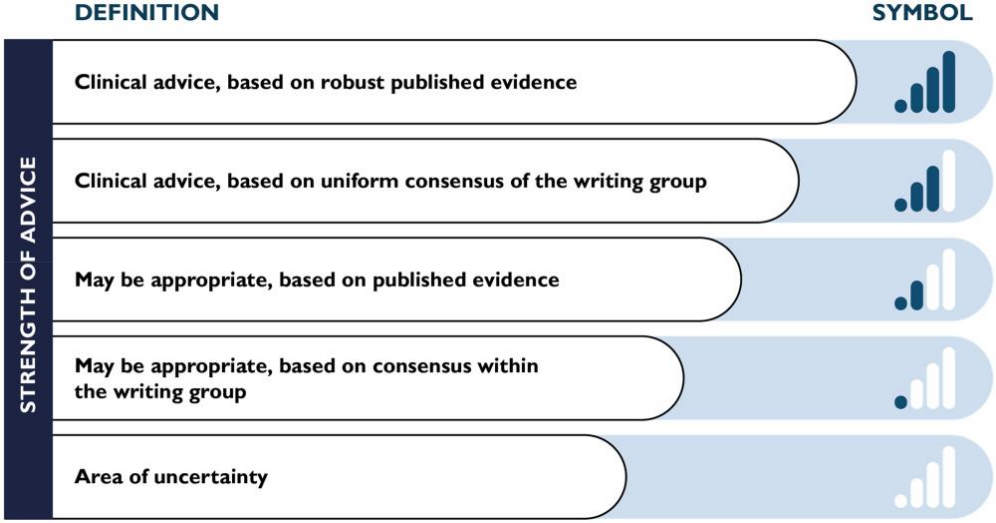
CMR, cardiac magnetic resonance; CPVT, catecholaminergic polymorphic ventricular tachycardia; ETT, exercise tolerance test; LQRSV, low QRS voltages; LV, left ventricular; PVC, premature ventricular contractions; TTE, transthoracic echocardiogram.

^a^Haemoglobin, thyroid function, and electrolytes.

**Table 3:** Risk stratification of PVCs in athletes

	Low-risk features	High-risk features
Clinical red flags		
Family history of premature SCD/cardiomyopathy	No	Yes
Suspicious symptoms	No	Yes
Physical examination	Normal	Abnormal
Resting ECG	Normal	Abnormal
Echocardiography	Normal	Abnormal
PVCs characteristics		
Morphology, number	Single	Multiple
Morphology, type	Fascicular (RBBB narrow QRS), infundibular (LBBB/inferior axis), RBBB/inferior axis (intermediate-risk)	RBBB/intermediate or superior axis LBBB/superior axis
Response to exercise	Suppression	Persistence/increase
R on T	No	Yes
NSVT	No	Yes

LBBB, left bundle branch block; RBBB, right bundle branch block; NSVT, non-sustained ventricular tachycardia; PVC, premature ventricular contraction.

In cases where first-line tests raise suspicion of cardiomyopathy, a CMR should be performed to verify the diagnosis. In athletes with isolated low QRS voltages and no abnormalities on TTE, ambulatory ECG monitoring and ETT, there is limited evidence to justify proceeding to CMR, which however can be considered if clinical suspicion remains, recognizing that, in our experience, it may occasionally reveal unexpected myocardial abnormalities not detected by other modalities. Asymptomatic athletes with normal evaluation may participate in all sports. Nevertheless, conducting annual surveillance assessments for athletes with low QRS voltages is advisable since the natural history of this anomaly is incomplete. First-line tests should be considered during the follow-up, with a low threshold for repeating a CMR with late gadolinium enhancement (LGE), if there are progressive ECG abnormalities and uncommon or complex ventricular extrasystoles during exercise testing.

### Premature ventricular contractions

Premature ventricular contractions pose a clinical challenge as they are relatively common in athletes, often stemming from increased activity of a benign electrical focus,^60^ and they may also signify underlying heart disease with a propensity for fatal arrhythmias and SCD. In athletes with PVCs on the ECG, it is crucial to strategize an appropriate diagnostic work-up to exclude serious cardiac conditions. This may be particularly challenging because certain ventricular arrhythmic substrates such as ACM, non-ischemic LV fibrosis and catecholaminergic polymorphic ventricular tachycardia (CPVT) may not be detected by first-line diagnostic tests (e.g. TTE). Generally, the diagnosis often necessitates more comprehensive investigations such as CMR, ETT and genetic testing^56,61,62^

In the past, the risk stratification of PVCs relied on the concept of ‘arrhythmic burden’, which suggested that a higher number of PVCs indicated a greater likelihood of an underlying cardiac condition. This perspective was primarily based on an observational study published in 2002 that found an association between the number of PVCs on 24-hour ECG monitoring and diagnostic yield of underlying cardiac disease.^62^ According to this concept, the 2017 International recommendations for interpretation of the athlete's ECG proposed further investigations only if two or more PVCs are present on a single 12-lead ECG reading.^6^ However, recent studies have questioned this approach by showing that consideration based primarily on the arrhythmic burden may not be accurate in detecting underlying cardiac conditions, lacking both sensitivity and specificity.^63,64^ Instead, the ventricular ectopic morphology, heterogeneity and complexity, coupling intervals and the relationship to exercise appear to offer a more accurate identification of an underlying cardiac disease^59,60,65^ (*Table 3*).

Analysis of the morphology of the PVCs is key for inferring their potential site of origin. Although benign PVCs can originate from multiple cardiac structures, the most common are the fascicles of the left bundle (typical in children and characterized by QRS duration < 130 ms and a typical incomplete RBBB configuration), the right ventricular outflow tract, the left ventricular outflow tract/aortic cusps and the anterior mitral annulus. Origin from these sites is associated with a lower probability of underlying structural heart disease, particularly in asymptomatic athletes with ‘isolated’ PVCs. Although most non-fascicular ectopy, such as right ventricular outflow tract (RVOT) variants, is benign even when frequent, certain features such as variable morphology, exercise augmentation, or atypical QRS characteristics may raise suspicion for underlying pathology and warrant further evaluation in selected cases. In contrast, PVCs with a broad RBBB pattern (QRS > 130 ms) and superior axis are uncommon among athletes. Recent studies have indicated an association between this PVC pattern and underlying LV scar. Additionally, if these PVCs fail to suppress during exercise, are reproduced during exercise testing/ambulatory ECG monitoring and/or display the ‘R on T’ phenomenon, they are more likely to be linked with LV scar.^60,66^

**Figure 4:**
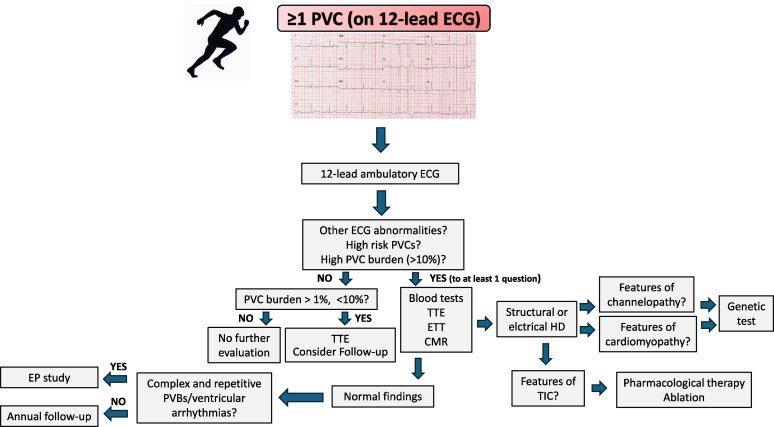
Management of athletes with PVCs. CMR, cardiac magnetic resonance; CTCA, computed tomography coronary angiography; D/C, discharge; EP, electrophysiological; ETT, exercise tolerance test; HD, heart disease; TIC, tachycardia-induced cardiomyopathy; TTE, transthoracic echocardiogram

Athletes displaying one or more PVCs on the resting ECG (with the exception of fascicular PVCs with RBBB QRS < 130 ms) should initially undergo ambulatory ECG monitoring for 24–48 h, preferably with a 12-lead configuration, including an exercise session to analyse the prevalence and morphology of the PVCs. Those with high-risk features (*Table 3*) should undergo further investigations, including a TTE (with coronary artery origin assessment), maximal ETT and CMR (*Figure 4*). Routine blood tests to assess haemoglobin, electrolyte levels and thyroid function and an assessment of cardiac structure and function through TTE and possibly with CMR is also advised in athletes with a PVC burden > 10%, irrespective of high-risk features, to exclude PVC-induced LV dysfunction.^67^ A CTCA may be considered *only* in athletes with high-risk PVCs and a clinical profile suggestive of coronary atherosclerosis (e.g. age > 35, cardiovascular risk factors, or concerning symptoms). An annual echocardiogram and exercise stress test are recommended in athletes with a high burden of extrasystoles who have had a normal comprehensive work-up to check for ventricular dysfunction and high-risk arrhythmias. When PVCs are polymorphic and exhibit distinct adrenergic dependency suggestive of CPVT, particularly after exclusion of structural heart disease, genetic testing for pathogenic variants implicated in CPVT should be considered.^68^ The clinical utility of employing invasive evaluation using an electrophysiological study incorporating programmed ventricular stimulation and endocardial voltage mapping in athletes with isolated high-risk PVCs/NSVT remains uncertain. This approach may be deemed appropriate in highly selected cases with complex ventricular arrhythmias or when non-invasive imaging suggests possible cardiomyopathy^69^ (*Table 2*).

If the comprehensive diagnostic work-up yields negative results, the athlete should be reassured but continue to remain under clinical surveillance if the arrhythmias are frequent or complex.

## Repolarization abnormalities

### T-wave inversion

T-wave inversion (TWI) is defined by a negative T wave ≥ 1 mm (0.1 mV) in depth in two or more contiguous leads (excluding leads aVR, III, and V1). TWI is one of the most common ECG manifestations in individuals with cardiomyopathies, including hypertrophic, dilated and arrhythmogenic cardiomyopathies (HCM, DCM, and ACM).^55,70,71^ However, this repolarization pattern may be a marker of physiological adaptation to exercise in some athletes, especially when present in the anterior leads and preceded by J-point/coved ST-segment elevation. The clinical significance of TWI varies according to its lead distribution on the ECG, namely anterior (leads V2–V4), lateral (V5 and/or V6, I and aVL) and inferior (II and aVF) leads. TWI may occasionally be more widespread, affecting the anterolateral and/or inferolateral leads.

#### Anterior T-wave inversion

Anterior TWI is relatively common in pre-pubertal individuals. However, with the progression of pubertal development, T waves typically become positive. Consequently, the prevalence of persistent TWI in V1–V3 falls to < 1% in athletes aged 16 years or older.^72–74^ According to the International recommendations for ECG interpretation in athletes, 16 years is considered the age cut-off for differentiating between benign (‘juvenile’) and potentially pathological TWI in V1–V3.^6^ However, in the age group 13–15 years, the persistence of TWI may still be considered potentially abnormal if pubertal development has already been completed.^72^ When there is doubt about the clinical significance of anterior TWI in the peri-pubertal age, reviewing prior ECG traces, if available, may be useful as a demonstration of normal repolarization patterns in the past would not support a benign persistence of the juvenile TWI. If no previous ECG is available, annual follow-up with ECG to demonstrate its normalization may be prudent.

The presence of anterior TWI limited to V2 is relatively common in white female athletes, identified in ∼4% of cases.^75^ Anterior TWI is also more common in male and female endurance athletes compared with white athletes participating in other sporting disciplines.^76^ Although outcome data is limited, current experience suggests that isolated anterior TWI limited to V1–V2 in female athletes does not require further investigations, in the absence of cardiac symptoms or concerning family history and when not accompanied by other ECG abnormalities.

Anterior TWI extending to V4 is observed in up to 13% of black athletes^74^ and 5.7% of mixed-race athletes as opposed to just 1.5% of white athletes.^77^ In accordance with the International recommendations for ECG interpretation in athletes,^6^ anterior TWI in athletes of African or Afro-Caribbean origin (black athletes) may be considered an early repolarization variant when preceded by J-point elevation and an ascending convex ST-segment. This repolarization pattern has also been noted in a minority of white athletes.^78^ A study comparing anterior TWI in black and white healthy athletes and patients with HCM and ACM showed that among athletes with anterior TWI, the combination of J-point elevation ≥ 1 mm and TWI confined to leads V1 to V4 excluded cardiomyopathy, irrespective of ethnicity.^79^ Conversely, anterior TWI with minimal or absent J-point elevation (<1 mm) may represent an underlying cardiomyopathy. According to this study and current recommendations,^6^ black athletes with the early-repolarization variant consisting of J-point/ST-segment elevation and TWI confined to V1–V4, do not require further investigations. However, it should be noted that there is limited long-term outcome data related to this pattern, highlighting the need for additional research in this area. It is also important to exercise caution in distinguishing this early-repolarization variant from the ‘coved-type’ (type 1) Brugada ECG pattern, which displays ST-segment elevation at the J-point followed by a descending ST-segment (see Supplementary data online, *Figures S1* and *S2*).^80^ When anterior TWI (even when limited to V2) coexists with other potential ECG markers of pathology, such as S wave prolongation (≥55 ms) in V1–V2, low QRS voltages, and high-risk PVCs (*Table 3*), a more in-depth evaluation is required to exclude an underlying cardiomyopathy (as per prior paragraphs) (*Table 4*).^6,55^

**Table 4:** Recommendations for diagnostic work-up of athletes with isolated TWI

	Degree of recommendation
Additional investigations are not usually required in athletes with isolated TWI in the anterior leads: When confined to V1–V2 in the absence of other suspicious clinical or electrocardiographic findings; When confined to V1–V3 in athletes < 16 years of age; When confined to V1–V4 and preceded by J-point/ST-segment elevation in black athletes.	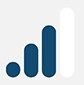
In athletes with anterior TWI (excluding scenarios listed above) or TWI in the lateral leads V4–V6, a comprehensive clinical work-up including TTE, maximal ETT, ambulatory ECG monitoring and CMR to exclude an underlying heart disease is strongly advised.	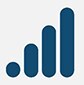
In adolescents aged 13–15, TWI in V1–V3 may require additional investigations if the pubertal development appears completed. If available, a previous ECG showing a positive anterior T wave reinforces the suspicion of a pathological pattern.	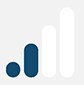
Although the clinical significance of isolated TWI in the inferior leads is unclear, investigation with TTE may be appropriate.	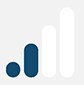
In athletes with inferior TWI, a CMR may be appropriate if the T waves are deep (≥0.2 mV) or preceded by ST depression, even if TTE does not reveal any positive findings.	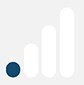
Genetic testing in athletes with isolated TWI, negative family history and a structurally normal heart is not advised.	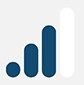

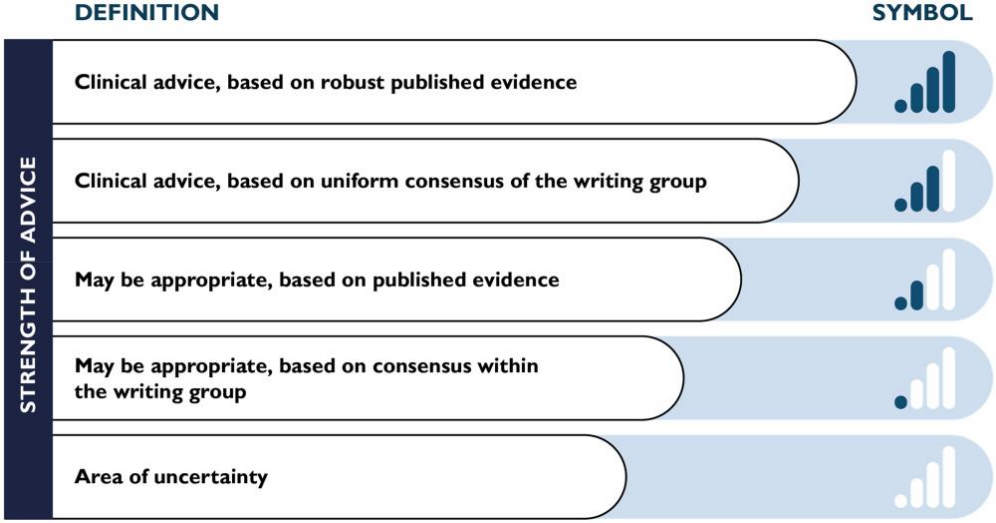
CMR, cardiac magnetic resonance; ETT, exercise tolerance test; TTE, transthoracic echocardiogram; TWI, T-wave inversion.

#### Lateral T-wave inversion—including anterolateral and inferolateral T-wave inversion

Lateral TWI is commonly detected in individuals with cardiomyopathy and is associated with a high diagnostic yield of cardiomyopathy or myocardial scar. It is well established that athletes displaying this ECG repolarization pattern should be investigated with CMR, ETT and prolonged ECG monitor.^47,70,81^ As per the International recommendations for ECG interpretation in athletes,^6^ in the absence of overt structural abnormalities or of an unifying diagnosis, annual echocardiographic surveillance is recommended, especially if the athlete is young (<35 years old), and serial CMR re-evaluation should be conducted every 2–4 years as lateral TWI may precede full phenotypic expression of cardiomyopathy by several years, particularly HCM^70^ (*Table 4*). Investigation with a CMR is advised if the annual surveillance reveals ECG or echocardiographic changes suggestive of a developing cardiomyopathy phenotype (for example, increase in the extent of the TWI, development or worsening of ST depression, arrhythmias at the ECG, or increase in wall thickness, diastolic dysfunction, LV obstructions at TTE).

It is important to note that the repolarization pattern in question is notably more prevalent in black athletes than in their white counterparts, with a frequency that is 10 times higher.^82^ However, the likelihood of diagnosing cardiomyopathy or myocardial scar is three times lower in black athletes compared to white athletes (19% vs 56%).^82^ Despite a compelling rationale for investigating all athletes with this repolarization anomaly, these statistics highlight the need for further research to pinpoint specific characteristics of TWI (such as depth, duration, pattern, and concomitant fragmentation) and customize investigations for greater efficacy in this demographic.

#### Inferior T-wave inversion

The significance of inferior TWI (lead II and aVF) is less clear than lateral TWI. It has been described in 6% of healthy black athletes and 1.5% of white athletes but also as an isolated finding in 1.9% of patients with HCM.^74^ Studies in black athletes have revealed a low diagnostic yield in athletes displaying inferior TWI in isolation. Nevertheless, conducting a TTE as the first test for all athletes is advisable until more extensive multicentric studies can ascertain the precise significance of inferior TWI. A CMR may be appropriate in asymptomatic athletes with deep T wave (≥0.2 mV) inversion or concomitant ST depression in the affected leads (*Table 4*).

**Figure 5:**
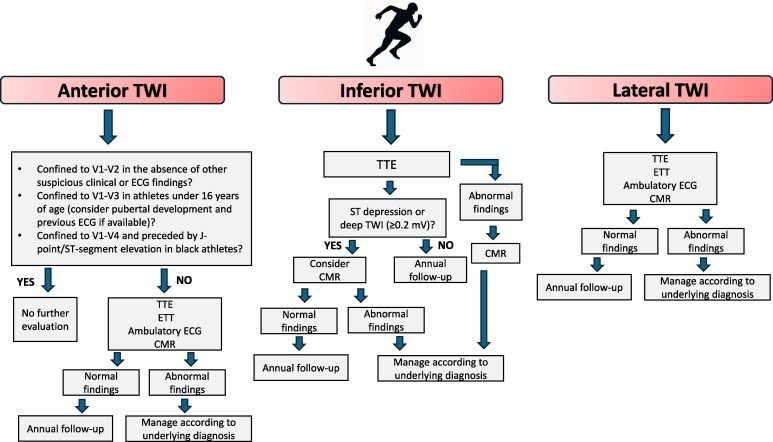
Management of athletes with TWI. CMR, cardiac magnetic resonance; CTCA, computed tomography coronary angiography; D/C, discharge; ETT, exercise tolerance test; HD, heart disease; TTE, transthoracic echocardiogram

*Figure 5* summarizes the diagnostic work-up of athletes with TWI. The presence of TWI in the absence of structural abnormalities does not warrant discouragement from participation in vigorous exercise and competitive sports. However, regular follow-up is advised. Familial evaluation of first-degree family members should be considered in young athletes with apparently unexplained TWI as the expression of cardiomyopathies varies with age and may not manifest until the fourth or fifth decade. However, genetic testing is not advised in TWI unless a specific phenotype is present or there is a family history of cardiomyopathy.^82,83^

### Type 2 and 3 Brugada patterns

Brugada syndrome (BrS) is an inherited cardiac ion channel disorder, most commonly affecting the sodium channels and is characterized by a predisposition to ventricular arrhythmias and SCD.^84^ There are three recognized Brugada ECG patterns (BrP), with only the type I pattern considered diagnostic and warranting investigation regardless of symptoms.^85^ Type 2 and Type 3 BrP describe J-point elevation (≥2 mm; 0.2 mV) and ascending saddle-shaped ST-segment elevation of variable height in leads V1 and/or V2 and ST-segment;^84^ in such cases, it is advisable to obtain an ECG with electrodes in high precordial position (second and/or third intercostal space) to potentially unmask a Brugada type I pattern^86^ (*Table 5*). Failure to demonstrate the type I BrS ECG pattern with this manoeuvre should reassure asymptomatic athletes. In selected cases, with type 2 or 3 BrP at baseline ECG and cardiac symptoms or family history of SCD at a young age, prolonged 12-lead ambulatory ECG monitoring using high right precordial lead positioning may aid in unmasking a diagnostic type 1 pattern^87–89^ (see Supplemental Figures).

**Table 5:** Diagnostic work-up of athletes with type 2 or 3 Brugada electrocardiogram patterns and borderline QT interval prolongation

	Degree of recommendation
In athletes with type 2 or 3 BrP, recording the ECG with electrodes in high precordial position (second and/or third intercostal space) should be performed to exclude a type 1 BrP.	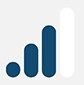
In asymptomatic athletes with type 2 or 3 BrP and no family history of sudden cardiac death or BrS, further investigations are not advised.	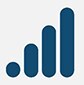
In athletes with QTc prolongation (>480 ms seen in repeated ECGs) a comprehensive clinical work-up including blood tests^a^, ETT, ambulatory ECG, TTE, genetic testing and evaluation of first-degree relatives’ ECG are strongly advised.	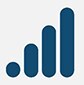
In athletes with borderline QTc (470–479 in females and 460–479 in males, seen in repeated ECGs) clinical evaluation with blood tests, ETT, ambulatory ECG is advised. Evaluation of first-degree relatives’ ECG (if possible) may facilitate the diagnosis. Genetic testing is justified only in the context of additional features suggestive of a LQTS diagnosis.	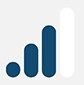
In athletes with borderline long QTc interval, negative clinical investigations and genetic testing, there is limited data that normalization of the QTc interval after 4–6 months may indicate exercise-induced prolongation.	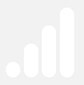

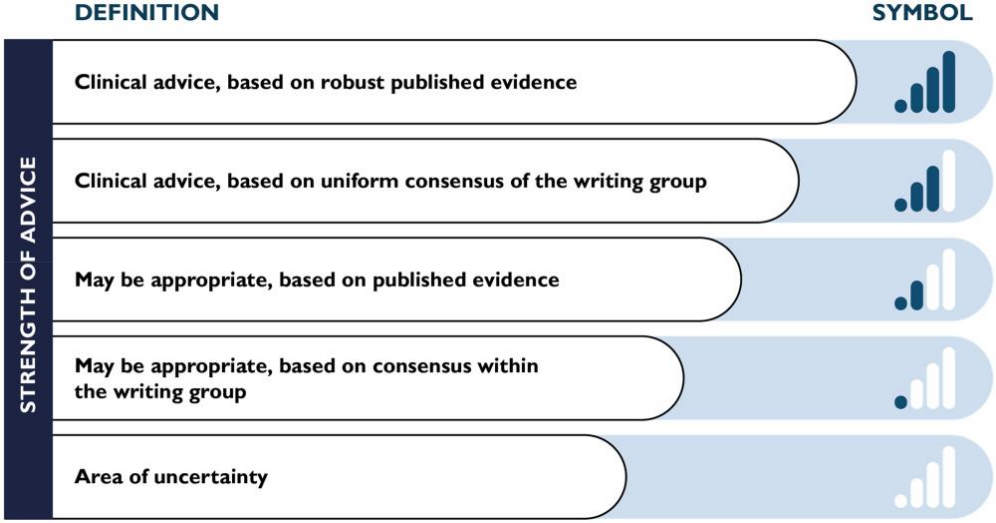
BrP, Brugada pattern; ETT, exercise tolerance test; LQTS, long QT syndrome; TTE, transthoracic echocardiogram.

^a^Haemoglobin, kidney function, and electrolytes.

### Borderline QTc interval

Long QT syndrome (LQTS) is characterized by QT interval prolongation and propensity to ventricular arrhythmias.^90^ Diagnosing LQTS can significantly impact athletes, affecting their participation in competitive sports and their risk of SCD. The measurement of the QTc interval in athletes can be challenging due to profound sinus bradycardia, sinus arrhythmia and prominent U waves, which can result in under-estimation or over-estimation of the QT interval.^91^ Accurate manual measurement of the QT interval is crucial, as described elsewhere.^6^ The probability of LQTS can be assessed using the modified Schwartz score derived from the 12-lead ECG, personal and family history, Holter monitoring and ETT.^92^ According to the ESC Sports Cardiology Guidelines, LQTS should be suspected if the corrected QTc interval, determined by Bazzett's formula, is ≥470 ms or ≥480 ms in asymptomatic male or female athletes, respectively, on a resting ECG or an ECG conducted 4 min into the recovery phase of an ETT.^93^ Additionally, as per the ESC Guidelines for the management of patients with ventricular arrhythmias and the prevention of SCD, a QTc ≥ 480 ms or an LQTS modified Schwartz score > 3 is diagnostic.^67^ The results of the Schwartz score in athletes may be sometimes difficult to interpret, since profound bradycardia, QTc > 460 ms and notched T waves occur in a small but significant proportion of athletes. Moreover, an accurate assessment of the 4 min recovery QT interval may be difficult due to upward ST shift, tall T waves and T-U complexes. Therefore, clinical suspicion is predominantly based on the resting ECG.

The detection of a prolonged QTc interval in an athlete should be confirmed at repeated ECG on a different day.

Asymptomatic athletes with a QTc > 470 ms for males or >480 ms for females should undergo a comprehensive diagnostic work-up, including a review of the drug history and the family history, assessment of electrolyte levels including potassium, calcium and magnesium, an ETT, ambulatory ECG (preferably with a 12-lead configuration to better assess the QT interval), TTE and genetic testing.^83^

Exercise tolerance testing plays a significant role in evaluating athletes with suspected LQTS, particularly in assessing paradoxical QT interval lengthening and identifying exertion-induced arrhythmias. We advise obtaining ECG printouts every 30 s for manual assessment of the QTc through all stages of the exercise test, although correction of the QT beyond a ventricular rate > 120 b.p.m. is challenging and inaccurate. Progressive prolongation of the QTc from a baseline QTc up to target heart rate and development of *de novo* T wave notching in at least three leads, are also valuable indicators of an underlying ion channel disorder.^94^

**Figure 6:**
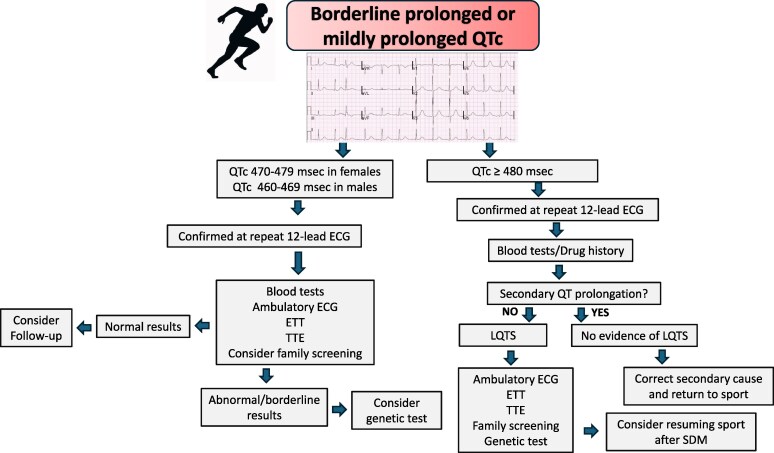
Management of athletes with borderline or mildly prolonged QTc interval. ETT, exercise tolerance test; LQTS, long QT syndrome; TTE, transthoracic echocardiogram; SDM, shared decision-making

Among athletes with borderline QTc prolongation (470–479 ms in females and 460–469 ms in males at repeated ECGs), blood tests, ambulatory ECG preferably with a 12-lead configuration, and ETT should be performed (*Figure 6*). If these tests yield normal results, annual surveillance should be considered, if there is a high index of suspicion, such as a personal history of syncope, a family history of SCD or LQTS. Genetic testing is justified only in the context of additional features suggestive of a LQTS diagnosis^83^ (*Table 5*).

Athletes with confirmed LQTS and a QTc > 480 ms may be considered for return to competitive sport following thorough evaluation, risk stratification, and implementation of individualized management strategies, including beta-blocker therapy, within a shared decision-making framework. Return to sport should also account for the LQTS genotype, prior symptoms, adherence to therapy, and appropriate emergency planning. Data suggesting an exercise-induced QTc prolongation and therefore a possible shortening after detraining are based on one scientific paper and should be corroborated by more robust evidence.^95^

## Conclusions

Electrical features recognized as abnormal and highly suggestive of cardiac disease may be isolated and unaccompanied by other pathological features supportive of a unifying diagnosis. Clinical management in these cases is often complex. Handling such cases can be intricate, as it is vital to strike a balance between avoiding unnecessary over-investigation and not overlooking potential issues. Recognized ECG patterns indicative of cardiac disease necessitate a comprehensive evaluation to exclude the broad spectrum of phenotypic manifestations of cardiac disease. When a definitive diagnosis is unattainable, a cautious approach should be adopted: in most instances, the athlete can continue to engage in competitive sports, but regular (annual) monitoring is advised. Additionally, evaluation of first-degree relatives may be appropriate in selected cases, particularly for young athletes whose underlying cardiac condition may not be fully expressed with ECG abnormalities potentially preceding complete phenotypic manifestation at an older age. Routine genetic testing is a powerful but imperfect tool, is generally not recommended for isolated ECG abnormalities and should be reserved for selected cases with a high index of suspicion for inherited disease.

Finally, the recommendations provided in this document are based on a careful review and synthesis of the existing sports cardiology literature coupled with the cumulative clinical experience of the writing group. It must be acknowledged that some of the recommendations herein provided lack rigorous scientific outcomes data and thereby represent important opportunities for future work.

## Supplementary Material

ehaf646_Supplementary_Data

## Declarations

### Data Availability

No data were generated or analysed for this manuscript.

### Pre-registered Clinical Trial Number

None supplied.
