# Correction to: Abnormal electrocardiogram findings in athletes: A consensus statement of the European Association of Preventive Cardiology of the European Society of Cardiology

**DOI:** 10.1093/eurheartj/ehaf1021

**Published:** 2025-12-15

This is a correction to: Gherardo Finocchiaro, Alessandro Zorzi, Mark Abela, Aaron Baggish, Silvia Castelletti, Elena Cavarretta, Guido Claessen, Domenico Corrado, Maria Sanz de la Garza, Sabiha Gati, Viviana Maestrini, Aneil Malhotra, Josef Niebauer, David Niederseer, Michael Papadakis, Antonio Pelliccia, Sanjay Sharma, Flavio D’Ascenzi, Abnormal electrocardiogram findings in athletes: A consensus statement of the European Association of Preventive Cardiology of the European Society of Cardiology, *European Heart Journal*, 2025, ehaf646, https://doi.org/10.1093/eurheartj/ehaf646

The originally published version of this manuscript erroneously omitted an affiliation for author Mark Abela. This error has been corrected.

