# Supplementary material for: Abnormal electrocardiogram findings in athletes: A consensus statement of the European Association of Preventive Cardiology of the European Society of Cardiology
Source: Eur Heart J. 2025 Sep 25;47(2):152–69. doi: 10.1093/eurheartj/ehaf646 (PMC12777699; doi:10.1093/eurheartj/ehaf646)
Supplement: Supplementary file 1 [file ehaf646_supplementary_data.zip › ehaf646_Supplementary_Data.odt]

Supplemental figures

Figure 1: saddle-back (type 2) Brugada ECG pattern with V1-V2 leads positioned in the 4th intercostal space (V1-V2). When V1-V2 leads are positioned in the 3rd intercostal space, a coved-type (type 1) Brugada ECG pattern is disclosed in V1.

A close-up of a graph

AI-generated content may be incorrect.

A close-up of a graph

AI-generated content may be incorrect.

B

A graph with lines on it

AI-generated content may be incorrect.Figure 2 – Differential diagnosis between type-1 Brugada ECG pattern (A), characterized by maximal displacement of the ST-segment at the J-point and the “black’s athlete” early repolarization variant (B) characterized by a domed ST-segment with maximal elevation after the J-point

A graph of a heart rate

AI-generated content may be incorrect.

A graph with lines on it

AI-generated content may be incorrect.

Figure 3 – Position of a 12 leads ambulatory ECG in an athlete with suspected Brugada pattern. All six precordial leads are positioned in the right and left parasternal lines, respectively in the 2nd (V1-V2), 3rd (V3-V4) and 4th (V5-V6) intercostal space. This configuration maximizes the possibility of disclosing the dynamic appearance of a type 1 Brugada ECG pattern.

A person with electrodes attached to his chest

AI-generated content may be incorrect.
